# Effect of Surgical Timing on the Refracture Rate after Percutaneous Vertebroplasty: A Retrospective Analysis of at Least 4-Year Follow-Up

**DOI:** 10.1155/2021/5503022

**Published:** 2021-11-27

**Authors:** Bin He, Jinqiu Zhao, Muzi Zhang, Guanyin Jiang, Ke Tang, Zhengxue Quan

**Affiliations:** ^1^Department of Orthopedics, The First Affiliated Hospital of Chongqing Medical University, Chongqing, China 400016; ^2^Department of Infectious Diseases, The First Affiliated Hospital of Chongqing Medical University, Chongqing, China 400016

## Abstract

**Introduction:**

The effect of surgical timing on vertebral refracture rate and mortality remains elusive after percutaneous kyphoplasty (PKP) or percutaneous vertebroplasty (PVP), and we aim to assess the impact of surgical timing on vertebral refracture rate and mortality in patients undergoing percutaneous vertebroplasty.

**Methods:**

We did a retrospective cohort study of patients who underwent PKP or PVP because of osteoporotic vertebral compression fracture (OVCF) between April 1, 2014 and March 31, 2016. The primary outcome measure was the incidence of vertebral refracture. Secondary outcomes included the mortality and chronic back pain.

**Results:**

The rate of vertebral refracture was significantly lower in early surgical timing group than that in late surgical timing group (HR 2.415, 95% CI 1.318–4.427; *P* = 0.004). We found that the bone mineral density (BMD) was only the risk factor to increase the vertebral refracture rate after vertebroplasty (*P* = 0.001). In addition, there was similar mortality between the two groups (15.7% in early surgical timing group versus 10% in late surgical timing group). Male patients (27.3%, 12/44) had higher mortality compared to female patients (10.6%, 20/189), while the mortality was higher in patients with cerebral infarction (25%, 3/12) than those without cerebral infarction (12.1%, 17/140).

**Conclusions:**

Surgical timing significantly affects the vertebral refracture rate after PKP or PVP, which is also influenced by BMD. The mortality after the surgery is not affected by the surgical timing, but gender and cerebral infarction may be the risk factors of mortality.

## 1. Introduction

Osteoporotic vertebral compression fracture (OVCF) widely occurs in elderly patients and results in back pain, spinal deformity, functional disability, and significantly reduced quality of life [1–4]. Conservative treatment needs relatively long time of bedrest, which increases the risk of pneumonia, pressure ulcers, and deep vein thrombosis, and even accounts for approximately 50% of fracture-related deaths [5–8]. Some patients have clinical sequelae due to the failure of fracture healing and persistent pain caused by delayed synostosis and pseudoarthrosis [8–11].

Percutaneous vertebroplasty (PVP) and percutaneous kyphoplasty (PKP) have become the leading treatment approaches for OVCF and are aimed at achieving pain relief, vertebral height restoration, and stability [4, 12–14]. These minimal invasive surgeries can reduce mortality [15, 16] and readmission rates in elderly patients [17]. Many patients who initially select nonstandard conservative treatment have the obviously increased risk of vertebral compression and sagittal deformity [7, 18, 19]. Sagittal deformity is associated with chronic pain and dysfunction. Then, vertebral cement augmentation surgeries may provide little improvement in the outcomes of patients after the failure of conservative treatment [8, 20].

Few studies explored the effect of surgical timing on the outcomes of OVCF. Early operation was documented to yield better correction of local spinal kyphosis, better alignment and back pain relief, and reduced rate of subsequent fracture than late operation [20, 21]. However, another study involving 115 patients revealed that vertebroplasty could obtain an immediate and sustainable improvement in the level of back pain and health-related quality of life, which was independent of the time from fracture (ranging from two to 12 months) [22].

The optimal surgical timing of PKP or PVP for treating OVCF remains controversial. This retrospective study is intended to explore the impact of surgical timing on the vertebral refracture rate and mortality in patients undergoing PKP or PVP due to OVCF. We also studied the influence of other risk factors (e.g., sex, age, and operative type) on these two important outcomes.

## 2. Methods

### 2.1. Data Sources and Study Population

We did a retrospective cohort study using validated data from The First Affiliated Hospital of Chongqing Medical University. The study was approved by the Ethics Committee of The First Affiliated Hospital of Chongqing Medical University and registered in the Chinese Clinical Trial Registry (ChiCTR2000032973). The database contained the records for demographic characteristics of all patients.

We included the patients who underwent PKP or PVP because of OVCF, and the surgery was conducted between April 1, 2014 and March 31, 2016. Patients with a previous history of internal fixation surgery were excluded. Postoperative medical management protocol for osteoporosis included caltrate, alfacalcidol, and bisphosphonates (alendronate or zoledronic acid) in standard doses. All patients were followed until June 10, 2020.

### 2.2. Outcomes

We recorded the baseline characteristics of each patient, and they included the age, gender, weight, height, body mass index (BMI), operation type (i.e., PKP, PVP, or PKP plus PVP), vertebral number of operation, pattern of cement, cement leakage, bone mineral density (BMD), smoking, drinking, hypertension, diabetes, coronary heart disease, cerebral infarction, lung diseases, and surgical timing. Patterns of cement were divided into connected and separated cement in the vertebral body. Cement leakage included intradiscal and paravertebral types. Surgical timing represented the time period from the injury to surgery.

The primary outcome was the incidence of vertebral refracture. Vertebral refracture was diagnosed by clinical symptoms (such as back pain) and magnetic resonance imaging examination. Our secondary outcomes were the mortality and back pain evaluated by Visual Analogue Scale (VAS). Given the influence of multiple surgeries with different surgical timing on the mortality and back pain, these patients who underwent multiple PKP or PVP were excluded. Patients were divided into two groups based on surgical timing: early surgical timing (≤21 days) and late surgical timing (>21 days).

### 2.3. Statistical Analysis

The data were expressed as mean (standard deviation, SD) or number. Statistical analyses were performed using independent *T*-test or Chi-square test, as appropriate. We used Kaplan-Meier analyses to assess the differences in the refracture rate and mortality. Early surgical timing and late surgical timing were compared with random-effects Cox proportional-hazards models. Univariate and multivariate logistic regression analyses were also performed to evaluate the risk factors of the refracture rate and mortality.

Descriptive statistical analyses were done using SPSS software (version 22.0). *P* value of less than 0.05 indicated a statistically significant difference.

### 2.4. Role of the Funding Source

The funders of this study had an important role in study design, data collection, data analysis, data interpretation, and writing of the report. The corresponding authors had full access to all the data in the study and had final responsibility for the decision to submit for publication.

## 3. Results

Between April 1, 2014 and March 31, 2016, 190 patients had PKP or PVP surgery due to OVCF.  Table 1 showed the baseline characteristics of both groups. There were 37 male and 153 female patients. The mean age of patients were 73.14 (SD, 8.49) years in the early surgical timing group and 71.21 (SD, 9.83) years in the late surgical timing group. The mean follow-up duration was 52.6 (SD 20.0) months.

No clinically relevant differences of baseline characteristics were identified between the two groups. Overall, 22.6% of patients suffered from symptomatic and vertebral refracture and 13.7% of patients died during the 4-6-year follow-up. The mean time of vertebral refracture was 27.6 (SD, 19.8) months and 29.3% of vertebral refracture occurred after 12 months of surgery.

**Figure 1:**
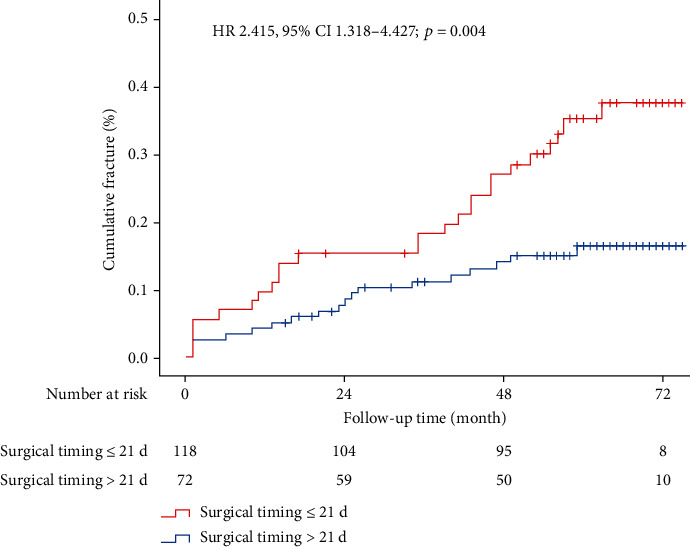
Vertebral refracture after early surgical timing versus late surgical timing. Kaplan-Meier estimates are from the overall pooled patient population.

Overall, 17 (14.4%) of 118 patients had symptomatic and vertebral refracture in the early surgical timing group, which were in contrast to 24 (33.3%) of 72 patients in the late surgical timing group during the 4-6-year period. 5 patients (29.4%) in the early surgical timing group and 7 patients (29.2%) in the late surgical timing group suffered from vertebral refracture after 12 months of surgery. The rate of vertebral refracture was significantly lower in the early surgical timing group than that in the late surgical timing group during the 4-6-year period (HR 2.415, 95% CI 1.318–4.427; *P* = 0.004; Figure 1).

Univariate and multivariate logistic regression analyses found that low BMD was the significant risk factor of vertebral refracture (univariate *P* = 0.001, multivariate *P* = 0.001; Table 2). In order to further assess the effect of BMD on the vertebral refracture rate, patients were divided into the low BMD group (*T* ≥ −3.5) and high BMD group (*T* < −3.5), and their rate of vertebral refracture were 42.6% (20/47) and 12.5% (16/128), respectively. These also confirmed that low BMD could significantly increase the incidence of vertebral refracture (Chi‐square = 19.002, *P* = 0.00001). Leakage of the bone cement was one of the most common complications of PKP or PVP, and intradiscal cement leakage had the trend of higher incidence of vertebral refracture (32.4%, 11/34) than no cement leakage (20.8%, 27/130), but with no statistical difference. Paravertebral cement leakage did not increase the incidence of vertebral refracture (12.5%, 3/24) compared to no cement leakage.

**Figure 2:**
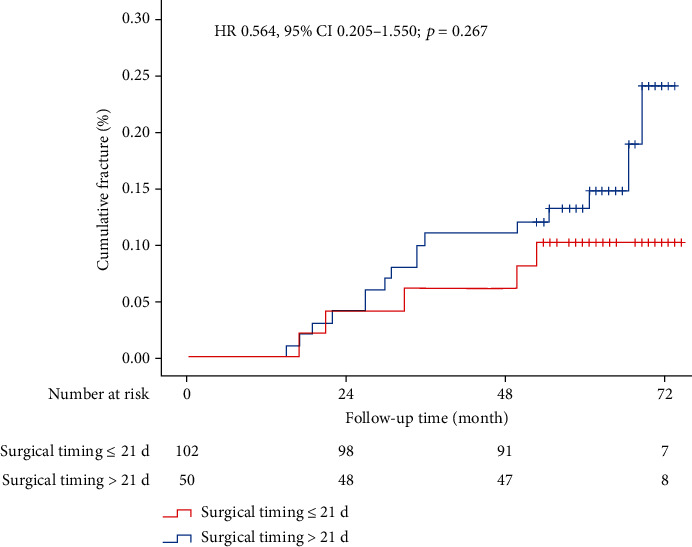
Mortality after early surgical timing versus late surgical timing. Kaplan-Meier estimates are from the overall pooled patient population.

After excluding the patients with more than 2 times of PKP or PVP, there were 16 deaths among 102 cases in the early surgical timing group (15.7%) and 5 deaths among 50 cases (10%) in the late surgical timing group. The mortality showed no statistical difference between the two groups (HR 0.564, 95% CI 0.205–1.550; *P* = 0.267; Figure 2). When comparing the two groups, gender (univariate *P* = 0.012, multivariate *P* = 0.012) and cerebral infarction (univariate *P* = 0.006, multivariate *P* = 0.006) were the significant factors that affected the mortality (Table 3). Male patients (25%, 8/32) showed higher mortality compared to female patients (10.8%, 13/120), while the mortality was higher in patients with cerebral infarction (25%, 3/12) than those without cerebral infarction (12.1%, 17/140). Other factors (i.e., age, weight, height, BMI, operation type, vertebral number, pattern of cement, cement leakage, BMD, smoking, drinking, hypertension, diabetes, coronary heart disease, and lung diseases) showed no obvious impact on the mortality (*P* > 0.05). Additionally, similar VAS scores remained between the early surgical timing group and late surgical timing group (1.43 ± 1.98 versus 1.51 ± 2.01, *P* = 0.79).

## 4. Discussion

To the best of our knowledge, this is the first clinical study to explore the effect of surgical timing (≤21 days versus >21 days) on the vertebral refracture rate and mortality after PKP or PVP during the 4-6-year follow-up. The results of this study confirm that early surgical timing (≤21 days) significantly reduces the rate of vertebral refracture than late surgical timing (>21 days), but similar mortality is observed between the two groups in patients undergoing PKP or PVP. In addition, low BMD is found to be the significant risk factor of vertebral refracture, while male sex and cerebral infarction substantially increase the mortality after PKP or PVP.

The aged population commonly suffers from OVCF which causes serious pain and remarkably reduced quality of life [23, 24]. Conservative treatment, PVP, and PKP are used to treat these patients. Many patients select the conservative treatment as the first choice because of delayed diagnosis, insufficient recognition, and economic problem. These lead to the delay of PKP and PVP which can immediately achieve pain relief and functional improvement [5, 25, 26] One recent meta-analysis compared nonsurgical treatment and PVP with PKP for treating OVCF and revealed that PKP was associated with significantly improved pain relief, functional status, and quality of life, accompanied by decreased risk of vertebral refracture [27].

Little is known about the optimal timing of cement augmentation surgeries. In one retrospective study, clinical and radiographic outcomes were compared based on different surgical timing of cement augmentation (operation within 4 weeks versus that later than 4 weeks). The results found that VAS and Oswestry Disability Index (ODI) were reduced significantly after PKP, but no difference was observed between early and late PKP [28]. These were consistent with Guan et al.'s study (operation within 2 weeks versus 2–4 weeks) [29] and Erkan et al.'s study (operation within 10 weeks versus later than 16 weeks) [30]. These similar improvements in VAS and ODI might be attributed by the fact that cement could immobilize the micromovement and destroy the terminal nerve endings in the fractured vertebral body [31, 32]. The chronic pain in these patients during the follow-up time of 4-6 years was also confirmed to be similar between early surgical timing and late surgical timing based on the results of our study.

However, only few studies explored the effect of surgical timing on the vertebral refracture rate after PKP or PVP, and no trials studied the mortality. One retrospective study only involved 62 patients who underwent PKP for OVCF and compared operation within 4 weeks with that later than 4 weeks. The results revealed the benefits of early PKP to reduce the refracture rate compared to late PKP (3/36 in early group versus 9/26 in late group, *P* < 0.01) at the follow-up of 6 months [28]. 124 patients with vertebroplasty were followed for at least 1 year, and PVP performed within 30 days was found to possibly reduce adjacent fracture in the thoracolumbar region [33]. Another retrospective study included 32 patients in the early group (<4 weeks) and 19 patients in the late group (>4 weeks), and the mean follow-up was 1.2 years. Early PKP revealed the reduced rates of subsequent fracture than late PKP [20]. These studies commonly had two limitations. Firstly, the sample size was relatively small. Secondly, the follow-up time was too short, which may not fully support the conclusion.

In this current study, 118 patients in the early surgical timing group (≤21 days) and 72 patients in the late surgical timing group (>21 days) were included, and the follow-up time was at least 4 years. The mean follow-up duration was 52.6 (SD 20.0) months. We divided all patients into two groups based on 21 days of surgical timing, because it was widely accepted to define fresh fracture and chronic fracture with specific value of 21 days after the injury. Kaplan-Meier estimates of vertebral refracture confirmed that early surgical timing had significantly reduced the refracture rate after PKP or PVP during the follow-up of 4-6 years (HR 2.415, 95% CI 1.318–4.427; *P* = 0.004).

In the early stage of fracture, patients might ignore their fracture and perform daily life walk before they were diagnosed with vertebral fracture, while in the late stage healing after fracture, local tissue organization impeded the even distribution of bone cement. These two factors resulted in the collapse of vertebral height and the progression of kyphosis [28]. Therefore, the early surgical timing of surgery benefited to improve the maintenance and restoration of vertebral body height and sagittal alignment. Additionally, we found that low BMD was a significant risk factor in increasing the incidence of vertebral refracture. Antiosteoporosis treatments are crucial to improve BMD and reduce the vertebral refracture rate in these patients with OVCF. Many drugs have been developed for antiosteoporosis treatments and mainly include calcitonin, teriparatide, bisphosphonates, and denosumab [34]. For instance, in the HORIZON Pivotal Fracture Trial involving patients with osteoporosis, bisphosphonate zoledronic acid was revealed to significantly improve BMD and reduce the risk of fracture [35]. One meta-analysis compared teriparatide versus bisphosphonates for treatment of osteoporosis, and the results found that bone forming agent teriparatide provided better promotion to improve BMD and reduce the risk of vertebral fracture in patients with osteoporosis than bisphosphonates [36]. These suggest that bone-forming agents such as teriparatide should be recommended for patients with OVCF.

Moreover, we first revealed that surgical timing showed no obvious impact on mortality after PKP or PVP due to OVCF (HR 0.564, 95% CI 0.205–1.550; *P* = 0.267), although early surgical timing could significantly reduce the vertebral refracture rate after vertebral cement augmentation surgeries. The possible explanation is that the most of these patients with vertebral refracture select PKP or PVP, and they do not need long time of bedrest that may increase the mortality. Additionally, male sex and cerebral infarction could substantially increase the mortality in these patients. In a nationwide register-based cohort study regarding hip fracture, long-term survival analyses revealed that strongly higher mortality for men was seen compared to women (HR 1.70, 95% CI 1.65-1.75, *P* < 0.001) [37]. This finding may be associated with stroke or other underdiagnosed diseases in male patients [38, 39].

We also should consider some limitations. Firstly, this is a retrospective study with inherent limitations due to the study design, although the patients in two groups have similar baseline characteristics. Secondly, the actual time of vertebral fracture may be not accurately established based on the patients' symptoms in some cases. Thirdly, some patients with multiple refracture and surgeries were excluded, which may alter the results of actual incidence of vertebral refracture and mortality.

## 5. Conclusion

Early surgical timing is associated with significantly reduced vertebral refracture rate than late surgical timing in patients undergoing PKP or PVP. Low BMD is confirmed to increase vertebral refracture rate, while male sex and cerebral infarction are revealed to increase the mortality in these patients.

## Figures and Tables

**Table 1:** Demographic data and clinical characteristics.

	Surgical timing ≤ 21 d	Surgical timing > 21 d	*P* value
Number	118	72	
Age (year)	73.14(8.49)	71.21(9.83)	0.153
Sex (male/female)	25/93	12/60	0.588
Height (cm)	155.07(6.37)	154.90(6.74)	0.866
Weight (kg)	55.74(9.17)	53.53(9.68)	0.115
BMI (kg/m^2^)	23.14(3.27)	22.29(3.67)	0.099
PKP/PVP/PKP+PVP	51/66/1	27/43/2	0.467
BMD (*T* scores)	2.60 (1.16)	2.77 (1.20)	0.340
Smoking	14	7	0.648
Drinking	13	5	0.352
Hypertension	47	23	0.274
Diabetes	15	10	0.816
Coronary heart disease	14	9	0.896
Cerebral infarction	8	4	0.736
Lung diseases	8	7	0.466

**Table 2:** Statistical analysis of risk factors for vertebral refracture.

Variable	Univariate *P* value	Multivariate *P* value
Age	0.092	0.092
Gender	0.951	0.951
Weight	0.625	0.625
Height	0.465	0.465
BMI	0.566	0.566
Operation type	0.709	0.709
Vertebral number	0.532	0.532
Pattern of cement	0.555	0.555
Cement leakage	0.764	0.764
BMD	0.001	0.001
Smoking	0.989	0.989
Drinking	0.998	—
Hypertension	0.586	0.586
Diabetes	0.766	0.766
Coronary heart disease	0.203	0.203
Cerebral infarction	0.330	0.330
Lung diseases	0.121	0.121

**Table 3:** Statistical analysis of risk factors for mortality.

Variable	Univariate *P* value	Multivariate *P* value
Age	0.509	0.509
Gender	0.012	0.012
Weight	0.284	0.284
Height	0.540	0.540
BMI	0.342	0.342
Operation type	0.230	0.230
Vertebral number	0.634	0.634
Pattern of cement	0.393	0.393
Cement leakage	0.986	0.986
BMD	0.794	0.794
Smoking	0.258	0.258
Drinking	0.269	0.269
Hypertension	0.569	0.569
Diabetes	0.072	0.072
Coronary heart disease	0.230	0.230
Cerebral infarction	0.006	0.006
Lung diseases	0.350	0.350

## Data Availability

The data generated during and/or analyzed during the current study are available after the reasonable application to the corresponding authors.
